# Socioeconomic disparities in prepregnancy BMI and impact on maternal and neonatal outcomes and postpartum weight retention: the EFHL longitudinal birth cohort study

**DOI:** 10.1186/1471-2393-14-314

**Published:** 2014-09-08

**Authors:** Shu-Kay Ng, Cate M Cameron, Andrew P Hills, Roderick J McClure, Paul A Scuffham

**Affiliations:** School of Medicine, Griffith Health Institute, Griffith University, Brisbane, QLD 4131 Australia; Centre of National Research on Disability and Rehabilitation, School of Human Services and Social Work, Griffith Health Institute, Griffith University, Brisbane, QLD 4131 Australia; Mater Mothers’ Hospital, Mater Research Institute – University of Queensland and Centre for Musculoskeletal Research, Griffith University, Brisbane, QLD Australia; Injury Research Institute, Monash University, Monash, VIC 3800 Australia

**Keywords:** Birth cohort, Obesity, Postpartum weight retention, Body mass index, Obstetric-neonatal outcome

## Abstract

**Background:**

Long-term obesity after pregnancy is associated with obesity prior to pregnancy and retention of weight postpartum. This study aims to identify socioeconomic differences in prepregnancy body mass index, quantify the impact of prepregnancy obesity on birth outcomes, and identify determinants of postpartum weight retention.

**Methods:**

A total of 2231 pregnant women, recruited from three public hospitals in Southeast Queensland in Australia during antenatal clinic visits, completed a questionnaire to elicit information on demographics, socioeconomic and behavioural characteristics. Perinatal information was extracted from hospital records. A follow-up questionnaire was completed by each participant at 12 months after the birth to obtain the mother’s postpartum weight, breastfeeding pattern, dietary and physical activity characteristics, and the child’s health and development information. Multivariate logistic regression method was used to model the association between prepregnancy obesity and outcomes.

**Results:**

Being overweight or obese prepregnancy was strongly associated with socioeconomic status and adverse behavioural factors. Obese women (18% of the cohort) were more likely to experience gestational diabetes, preeclampsia, cesarean delivery, and their children were more likely to experience intensive- or special-care nursery admission, fetal distress, resuscitation, and macrosomia. Women were more likely to retain weight postpartum if they consumed three or fewer serves of fruit/vegetables per day, did not engage in recreational activity with their baby, spent less than once a week on walking for 30 minutes or more or spent time with friends less than once per week. Mothers who breastfed for more than 3 months had reduced likelihood of high postpartum weight retention.

**Conclusions:**

Findings provide additional specificity to the increasing evidence of the predisposition of obesity prepregnancy on adverse maternal and perinatal outcomes. They may be used to target effective behavioural change interventions to address obesity in women.

## Background

In Australia, approximately one-third of all pregnant women are overweight or obese [1]. A similar prevalence of overweight and obesity among pregnant women has been observed in the United States [2]. In both countries the proportion of pregnant women who are overweight or obese is increasing [3, 4].

Pregnant women who are overweight or obese have a disproportionate risk of induced preterm delivery [5] and maternal, intrapartum, peripartum, neonatal [6, 7], and postpartum complications including gestational diabetes mellitus (GDM), type 2 diabetes, high blood pressure, dyslipidaemia, cardiovascular disease and several major cancers [7, 8]. The offspring of these women also have a significantly elevated risk of adverse short- and long-term health issues [9–11]. For example, children of women with GDM are more likely to be obese and have impaired glucose tolerance and diabetes in childhood and adulthood [12, 13].

Increased risk of being overweight after the first and subsequent pregnancies is associated with the level of obesity prior to pregnancy [14], gestational weight gain above the recommended guidelines [15, 16], and failure to lose gestational weight in a reasonable timeframe (excessive postpartum weight retention) [17]. Substantial evidence also links net weight gain after pregnancy to obesity in later life [18] and shows that women who fail to lose weight postpartum have a higher risk of subsequent long-term obesity [19].

It has been shown that adverse factors such as lack of nutrition knowledge [20, 21], poor dietary habits and physical inactivity [22, 23] could contribute to being overweight or obese during pregnancy as well as having high postpartum weight gain and/or retention. Findings from a recent retrospective cohort study [4] confirmed the commonly described association between maternal obesity, lower socioeconomic status [24, 25] and indicated the role of adverse health behaviours in explaining this socioeconomic status differential [26]. The importance of this finding relates to the potential for addressing the high prevalence of overweight and obesity among pregnant women through screening and targeted behaviour change interventions in high-risk groups. Interventions based on specific knowledge of the subgroups at greatest risk and the modifiable behavioural determinants would lead to substantial population benefit by interrupting the transgenerational repeating cycle of risk [25].

There is currently insufficient knowledge to generate and refine targeted public health interventions to reduce transgenerational obesity because population-based studies on the impact of obesity on birth outcomes are relatively scant, especially country-specific studies such as for Australia. The present study is a prospective and multi-year longitudinal birth cohort study, and collects a spectrum of eco-epidemiological factors [27]. It thus offers a unique opportunity to understand the various exposures that have impact on birth and postpartum outcomes. The aims of this study were to identify any socioeconomic differential in prepregnancy body mass index (BMI), to quantify the impact of prepregnancy obesity on maternal and neonatal outcomes, and to identify determinants that are associated with postpartum weight retention. The identification of the socioeconomic differential in prepregnancy obesity and the modifiable risk factors in excessive weight retention postpartum will be useful for targeting future behavioural change interventions, identifying population groups who would benefit from public health interventions, and promoting research in women’s health to address the problem of obesity.

## Methods

This prospective cohort study was conducted and reported in accordance with the STROBE guidelines ( http://www.strobe-statement.org/).

### Study design and subjects

The birth cohort "Environments for Healthy Living" (EFHL) is a population-based longitudinal study which commenced the pilot phase of recruitment in November 2006 and open recruitment in August 2007 to investigate the relationship between social, environmental and behavioural factors and the health and development of children in Southeast Queensland, Australia [27]. The study area contains an estimated population of over 1 300 000 people or approximately 30% of Queensland’s population. The study region is markedly heterogeneous with respect to socioeconomic distribution; in particular, the Health Districts of the study region are known to have higher proportions of socio-economic disadvantage than the national average [28, 29].

Women who planned to give birth at one of three participating hospitals were eligible to participate and enrol their baby in this study. Pregnant women aged less than 16 years or unable to provide informed consent were excluded [27]. Written informed consent was obtained for release of hospital perinatal data related to the birth of each child, completion of a participant baseline survey and for individual follow-up. During the first four open recruitment phases of the study (2007 to 2010), the total number of mothers approached was 5149, of whom 2254 women (43.8%) agreed to participate and 2277 babies have been registered with the study (including 23 sets of twins).

**Figure 1:**
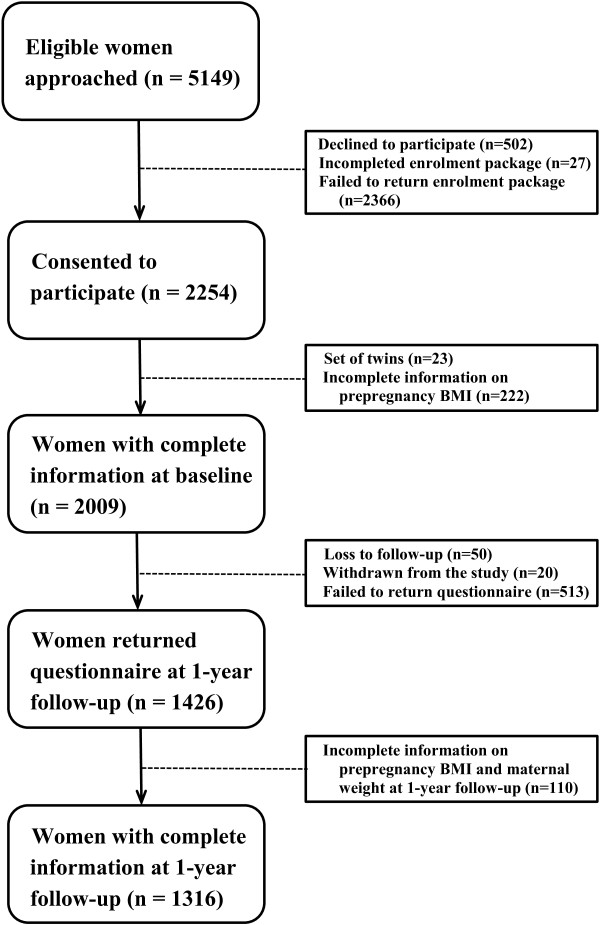
**Flow diagram of recruitment and loss to follow-up for the EFHL cohorts 2007 – 2010.**

Following recruitment, a questionnaire was completed by each mother to elicit baseline information on demographics, socioeconomic status, family structure, behavioural and pregnancy characteristics. Perinatal information was extracted from hospital birth records. Follow-up routinely occurs when each child reaches 1, 3, and 5 years of age [27]. Information on maternal physical activity, dietary intake and breastfeeding duration, familial and social exposures as well as child health was collected via self-report questionnaires. In this study, multiple births were excluded (n = 46), leaving 2231 mothers and babies at baseline. Of 2231 mothers, 2009 (90%) have complete information on prepregnancy BMI. At 1-year follow-up, 1426 mothers (63.9% of 2231) returned questionnaires. Of 1426 mothers, 1316 (92%) have complete information on prepregnancy BMI and maternal weight at 1-year follow up. Figure 1 presents a flow diagram of recruitment and loss to follow-up for the present study.

### Measurements

A wide variety of health-related exposures and outcomes variables were measured at baseline (self-report questionnaire and hospital birth record) and during the 1-year follow-up (self-report questionnaire) with variables classified under the following domains: (a) Maternal; (b) Nutrition and physical activity factors; (c) Household and family; (d) Pregnancy; and (e) Child factors.

Maternal characteristics measured at baseline included self-reported prepregnancy weight and height, place of birth, maternal age, education level, employment status, marital status, smoking, alcohol and ‘over the counter’ medications intake patterns for non-medical purposes during pregnancy, and psychological distress – measured using the Kessler-6 (K6) psychological distress scale. The K6 has been widely used and has demonstrated excellent internal consistency and reliability [30, 31]. Three levels of risk of psychological distress were considered on the basis of the overall score of the 6 items: Low-risk (0–7); Medium-risk (8–12); and High-risk (13+) [32]. Using the self-reported prepregnancy weight and height, prepregnancy BMI was classified on the basis of the World Health Organisation (WHO) criteria (underweight: <18.5 kg/m^2^; normal-weight: 18.5–24.9 kg/m^2^; overweight: 25.0–29.9 kg/m^2^; obese: ≥ 30 kg/m^2^) [33]. At 1-year follow-up maternal measurements included self-reported postpartum weight, psychological distress, frequency of 5 or more (5+) alcoholic drinks on one occasion (once per week or more often compared to less than once per week), having a paid job after birth, and frequency of spending time with family and friends. Postpartum weight retention was calculated by subtracting the prepregnancy weight from the current weight at 1-year follow-up.

Nutrition and physical activity factors measured at baseline are mineral and vitamin supplements taken prior to and during pregnancy (Iron, Zinc, Calcium, Folic-acid, Multivitamins, Vitamin-C, Vitamin-E, Pregnancy/Breastfeeding supplement). Factors measured at 1-year follow up included breastfeeding duration, fruit and vegetable intake, frequency of takeaways, attendance at recreational activities with baby (such as, play groups, mothers’ groups, or swimming classes), and the frequencies of moderate physical activities and walking (30 minutes or more).

Household and family factors were obtained at baseline (including partner’s education level and employment status, household ownership and number of children under 16 years of age) and during the follow-up period at 1-year (including the frequency of being a single parent for 1 month or longer, whether the mother is living with the biological father of the child, spouse or partner, whether their relationship status changed, environment with child exposure to passive smoking, and frequency of eating takeaway food).

Variables in the pregnancy domain were obtained from hospital birth record, which included parity, pre-existing hypertension, obstetric complications (hyperglycaemia, gestational diabetes, preeclampsia, cesarean delivery), and neonatal complications (fetal distress, admission to an intensive or special care nursery, jaundice, respiratory distress, congenital anomaly, resuscitation procedure).

Child characteristics included gender, birthweight, and at 1-year mother-reported weight gain, injury, and indication of health-related conditions (asthma, chest infection) diagnosed by a medical doctor. Macrosomia was defined as birthweight > 4 kg, irrespective of gestational age [34].

## Ethical approval

Approval for the study was obtained from the Griffith University Ethics Committee and the Human Research Ethics Review Committees of the three participating public maternity hospitals (Logan, Gold Coast and Tweed Hospitals) in the study area.

### Statistical analysis

Data were analysed using IBM SPSS-22 (IBM, Chicago, IL). Baseline characteristics of the EFHL cohort were compared with all births with gestation of 28 weeks or more in the study region between 2007 and 2010, in an attempt to address potential selection bias. Missing data including loss to follow-up were handled using a complete-case approach. Measures of association and ordinal association between prepregnancy BMI groups and categorical variables were obtained using Pearson’s chi-square and Gamma statistics, respectively. Comparisons of continuous variables between the four prepregnancy BMI groups were tested using one-way ANOVA (with Tukey’s post-hoc multiple test). Multiple logistic regression was performed to identify pregnancy and neonatal problems that associate with the prepregnancy underweight, overweight and obese mothers. Adjusted odds ratios (ORs) with 95% confidence intervals (CIs) were calculated after accounting for potential confounding factors that are significantly associated with the prepregnancy BMI groups. These include maternal age, employment and education, alcohol intake and smoking during pregnancy, non-medical drug use, primiparity, pre-existing hypertension, marital status, paternal employment and education, household ownership, and the number of children living in the household aged under 16 years. The year of recruitment was also considered as a potential confounder, given temporal differences were identified for some antenatal exposures [29]. Polynomial contrast within multiple logistic regression models were used to test the linear trend of adjusted ORs.

Cut-off points for high postpartum weight retention were determined separately for three prepregnancy BMI groups (normal, overweight, and obese) by the highest weight retention quintile. This group of women with high postpartum weight retention (weight retention > top quintile) thus contains about 20% of women in each of the three pregnancy BMI groups. Multiple logistic regression was conducted to identify risk factors at 12-month follow-up that are associated with high postpartum weight retention. Adjusted ORs with 95% CIs were calculated, after accounting for potential confounding factors including the year of recruitment, the prepregnancy BMI grouping, and those factors defined above.

## Results

### Sample characteristics

Baseline characteristics of the EFHL cohort are displayed in Table 1 along with corresponding details of births with gestation of 28 weeks or more in the study region between 2007–2010. The birth cohort sample did not differ significantly from the general population for gender, plurality, or birth outcome. However, our sample had a smaller proportion of mothers who were younger than 20 years of age and infants who were born between 28 and 36 weeks gestation. Moreover, the percentage of low birthweight infants (<2500 g) was approximately half that of babies born in the general population.

**Table 1:** **Baseline characteristics of the cohort and comparisons with all births in the study region (with gestational 28 weeks or more)**

Characteristics	Frequency (percentage ^a^)		
	Birth cohort sample (Years 2007 to 2010) 2254 women 2277 babies	Deliveries in region ^b^ (Years 2007 to 2010) 36620 women 37061 babies	***p***-value ^c^ (two-sided)
Maternal age			
< 20 years	107 (4.8%)	2559 (7.0%)	< 0.001
20-24 years	438 (19.9%)	6407 (17.6%)	
25-29 years	632 (28.6%)	10376 (28.5%)	
30-34 years	560 (25.4%)	10088 (27.7%)	
≥ 35 years	469 (21.3%)	6980 (19.2%)	
Missing data	48	0	
Gender of infant			
Male	1127 (50.3%)	18914 (51.3%)	0.324
Female	1115 (49.7%)	17926 (48.7%)	
Missing data	35	0	
Birthweight			
< 2500 g	55 (2.5%)	1800 (4.9%)	< 0.001
2500-3999 g	1809 (81.5%)	30207 (82.0%)	
≥ 4000 g	355 (16.0%)	4831 (13.1%)	
Missing data	58	2	
Gestational age at birth			
28-36 weeks	79 (3.5%)	2376 (6.4%)	< 0.001
37-41 weeks	2131 (95.9%)	34264 (93.0%)	
42 weeks	13 (0.6%)	200 (0.5%)	
Missing data	54	0	
Plurality			
Singleton	2207 (98.0%)	35980 (97.7%)	
Multiple	46 (2.0%)	860 (2.3%)	0.370
Missing data	24	0	
Outcome			
Live birth	2273 (99.8%)	36728 (99.7%)	
Stillbirth	4 (0.2%)	112 (0.3%)	0.274
Missing data	0	0	

^a^Percentages are calculated based on the available (non-missing) data.

^b^Data include only deliveries with gestational 28 weeks or more; data are provided by Queensland Health and New South Wales Health for the study region (Logan, Beaudesert, Gold Coast and Tweed).

^c^Chi-square test for comparing proportions between birth cohort sample and the general population.

### Prepregnancy BMI

The baseline descriptive characteristics of participants stratified by the prepregnancy BMI is shown in Table 2. Underweight women were younger compared to women with a normal prepregnancy BMI (reference group). On the other hand, compared to women with a normal prepregnancy BMI, overweight and obese women had lower education level. Their partners were more likely to be unemployed or not in the labour force, and had lower education level. Obese and underweight women were less likely to be married, own their own home, and consume alcohol during pregnancy. However, they were more likely to smoke during pregnancy and be engaged in non-medical drug use. Supplement intake prior to and during pregnancy was different between the four prepregnancy BMI groups. There were moderate negative ordinal relationships between intake of major supplements and prepregnancy BMI with obese women less likely to take supplements of all types considered in the study, especially iron, multivitamins, vitamins-C and E. There was a moderate positive ordinal relationship between number of children under 16 years of age in the household and prepregnancy BMI (0.170, *p* < 0.001), indicating that the proportion of prepregnancy obesity increases when the number of children under 16 years old increases. A moderate positive ordinal relationship between parity and prepregnancy BMI was also observed (0.201, *p* < 0.001), indicating that the proportion of prepregnancy obesity increases for mothers who have had previous pregnancies. The strong relationship between pre-existing hypertension and prepregnancy BMI (0.495, *p* = 0.021) confirms that obese women are more likely to have the pre-existing condition.

**Table 2:** **Baseline characteristics of participants stratified by the prepregnancy BMI (n = 2009)**

	Prepregnancy BMI				
	Underweight (BMI < 18.5)	Normal weight (BMI 18.5-24.99)	Overweight (BMI 25-29.99)	Obese (BMI ≥ 30)	
Characteristics	(N=167)	(N= 1052)	(N= 430)	(N= 360)	***p***-value (two-sided)
Maternal Characteristics:					
Maternal age (y)					
< 20	12 (7.3%)	48 (4.7%)	14 (3.3%)	11 (3.1%)	0.017*^b^
20-24	49 (29.7%)	198 (19.2%)	69 (16.3%)	74 (21.0%)	
25-29	38 (23.0%)	293 (28.4%)	135 (31.8%)	99 (28.0%)	
30-34	33 (20.0%)	280 (27.2%)	119 (28.1%)	89 (25.2%)	
≥ 35	33 (20.0%)	212 (20.6%)	87 (20.5%)	80 (22.7%)	
Missing data	2	21	6	7	
Born in Australia	114 (68.3%)	733 (69.7%)	312 (72.6%)	259 (71.9%)	0.589
Missing data	0	1	0	0	
Maternal employment					
Employed	75 (46.3%)	564 (54.5%)	209 (49.2%)	169 (48.3%)	0.038*^b^
Unemployed	26 (16.0%)	113 (10.9%)	42 (9.9%)	36 (10.3%)	
Not in-labour force	61 (37.7%)	358 (34.6%)	174 (40.9%)	145 (41.4%)	
Missing data	5	17	5	10	
Marital status					
Single	15 (9.0%)	65 (6.2%)	20 (4.7%)	31 (8.6%)	0.001*^b^
Dating relationship	14 (8.4%)	32 (3.1%)	17 (4.0%)	22 (6.1%)	
De facto	76 (45.5%)	382 (36.4%)	158 (37.0%)	126 (35.0%)	
Married	62 (37.1%)	557 (53.1%)	225 (52.7%)	175 (48.6%)	
Separate/Divorce/Widow	0 (0%)	13 (1.2%)	7 (1.6%)	6 (1.7%)	
Missing data	0	3	3	0	
Maternal education level					
Primary school	2 (1.2%)	9 (0.9%)	4 (0.9%)	4 (1.1%)	<0.001*^b^
Not complete secondary	37 (22.3%)	176 (16.8%)	85 (20.0%)	77 (21.5%)	
Secondary school	50 (30.1%)	301 (28.7%)	137 (32.2%)	129 (36.0%)	
TAFE/Trade	46 (27.7%)	306 (29.2%)	129 (30.3%)	107 (29.9%)	
University	31 (18.7%)	257 (24.5%)	71 (16.7%)	41 (11.5%)	
Missing data	1	3	4	2	
Nutrition Factors:					
Supplements Intake					
Iron	51 (30.5%)	301 (28.6%)	118 (27.4%)	67 (18.6%)	0.001*^a-^
Zinc	37 (23.3%)	196 (19.8%)	80 (19.2%)	53 (15.4%)	0.039*^a-^
Calcium	27 (16.2%)	139 (13.2%)	40 (9.3%)	32 (8.9%)	0.001*^a-^
Folic acid	84 (52.5%)	511 (51.5%)	222 (53.0%)	166 (47.7%)	0.499
Multivitamins	93 (55.7%)	548 (52.1%)	214 (49.8%)	140 (38.9%)	<0.001*^a-^
Vitamin C	55 (34.6%)	337 (34.1%)	133 (32.0%)	80 (23.2%)	0.001*^a-^
Vitamin E	113 (67.7%)	735 (69.9%)	280 (65.1%)	191 (53.1%)	<0.001*^a-^
Pregnancy/breastfeeding sup.	38 (23.9%)	193 (19.6%)	91 (21.9%)	40 (11.5%)	0.004*^a-^
Household and Family Structure:					
Partner’s employment					
Employed	142 (89.9%)	931 (93.7%)	392 (96.8%)	301 (90.7%)	0.017*^b^
Unemployed	10 (6.3%)	37 (3.7%)	7 (1.7%)	17 (5.1%)	
Not in-labour force	6 (3.8%)	26 (2.6%)	6 (1.5%)	14 (4.2%)	
Missing data	9	58	25	28	
Partner’s education level					
Primary school	4 (2.6%)	9 (0.9%)	5 (1.2%)	0 (0%)	<0.001*^b^
Not complete secondary	33 (21.2%)	182 (18.2%)	89 (21.9%)	94 (28.6%)	
Secondary school	41 (26.3%)	243 (24.3%)	114 (28.0%)	89 (27.1%)	
TAFE/Trade	52 (33.3%)	421 (42.2%)	150 (36.9%)	120 (36.5%)	
University	26 (16.7%)	143 (14.3%)	49 (12.0%)	26 (7.9%)	
Missing data	11	54	23	31	
Household ownership					
Own outright	6 (3.7%)	33 (3.2%)	3 (0.7%)	3 (0.8%)	<0.001*^b^
Own with mortgage	52 (31.9%)	457 (44.3%)	189 (44.9%)	121 (34.1%)	
Private rent	72 (44.2%)	446 (43.2%)	193 (45.8%)	178 (50.1%)	
Public housing	5 (3.1%)	23 (2.2%)	7 (1.7%)	13 (3.7%)	
Board with parents	28 (17.2%)	73 (7.1%)	29 (6.9%)	40 (11.3%)	
Missing data	4	20	9	5	
Number of children under 16 y (household)					
Nil	78 (47.3%)	413 (39.7%)	134 (31.6%)	115 (31.9%)	<0.001*^a+^
1-3	83 (50.3%)	595 (57.2%)	279 (65.8%)	217 (60.3%)	
More than 3	4 (2.4%)	33 (3.2%)	11 (2.6%)	28 (7.8%)	
Missing data	2	11	6	0	
Maternal Health and Behaviours During Pregnancy:					
Maternal psychological distress					0.343
Low risk	128 (77.6%)	853 (81.6%)	352 (83.0%)	280 (78.4%)	
Medium risk	30 (18.2%)	158 (15.1%)	64 (15.1%)	61 (17.1%)	
High risk	7 (4.2%)	34 (3.3%)	8 (1.9%)	16 (4.5%)	
Missing data	2	7	6	3	
Smoke during pregnancy	59 (35.3%)	233 (22.2%)	109 (25.5%)	108 (30.3%)	<0.001*^b^
Missing data	0	1	3	3	
Alcohol intake	69 (41.3%)	536 (51.0%)	202 (47.4%)	137 (38.3%)	0.001*^b^
Missing data	0	0	4	2	
Non-medical drug use^a^	29 (17.6%)	98 (9.5%)	35 (8.3%)	42 (11.8%)	0.005*^b^
Missing data	2	21	8	3	
Birth-related Factors:					
Primiparity	97 (58.8%)	674 (64.8%)	313 (73.5%)	267 (75.2%)	<0.001*^a+^
Missing data	2	12	4	5	
Baby gender (Male)	80 (48.5%)	527 (50.9%)	201 (47.2%)	182 (51.3%)	0.564
Missing data	2	16	4	5	
Pre-existing hypertension	0 (0%)	6 (0.7%)	3 (0.8%)	8 (2.4%)	0.021*^a+^
Missing data	23	144	48	26	

^*^Significant at the 0.05 level.

^a^Test for ordinal association using Gamma coefficient (^+^indicates a positive association; ^-^indicates a negative association).

^b^Test for association using chi-square test.

### Pregnancy and neonatal outcomes

The impact of prepregnancy obesity on pregnancy and neonatal outcomes after adjusting for confounders is presented in Table 3. With an increasing level of obesity prior to pregnancy, a linear trend of increasing risk of adverse outcomes was observed for hyperglycaemia (*p* for trend = 0.024), cesarean delivery (*p* for trend < 0.001), intensive or special care nursery admission (*p* for trend < 0.001), resuscitation (*p* for trend = 0.001), and macrosomia (*p* for trend < 0.001). In addition to these adverse outcomes, obese women were also more likely to have GDM (adjusted OR = 2.327) and preeclampsia (adjusted OR = 3.143). Further, their newborns had also a higher chance of having fetal distress (adjusted OR = 1.870). Babies of overweight women were also more likely to have jaundice (adjusted OR = 1.762).

**Table 3:** **Impact of prepregnancy obesity on pregnancy and neonatal outcomes (n = 2009)**

Outcome	Frequency (%)	Adjusted OR ^a^ (95% CI)	***p***-value (2-sided)
Obstetric Complication:			
Hyperglycaemia			
Normal weight	65 (8.0%)	Reference	
Underweight	5 (3.7%)	0.532 (0.207 – 1.371)	0.192
Overweight	35 (9.7%)	1.210 (0.754 – 1.940)	0.430
Obesity	38 (11.8%)	1.464 (0.921 – 2.328)	0.107
Missing^b^	382		Trend^c^ (0.024*)
Gestational diabetes			
Normal weight	21 (2.4%)	Reference	
Underweight	0 (0%)	—	—
Overweight	7 (2.0%)	1.040 (0.396 – 2.732)	0.937
Obesity	11 (3.6%)	2.327 (1.037 – 5.223)	0.041*
Missing	337		
Preeclampsia			
Normal weight	25 (3.1%)	Reference	
Underweight	0 (0%)	—	—
Overweight	11 (3.3%)	1.184 (0.559 – 2.505)	0.660
Obesity	29 (9.8%)	3.143 (1.694 – 5.830)	< 0.001*
Missing	430		
Cesarean delivery			
Normal weight	238 (23.0%)	Reference	
Underweight	38 (23.0%)	0.950 (0.588 – 1.536)	0.834
Overweight	146 (34.4%)	1.802 (1.349 – 2.409)	<0.001*
Obesity	127 (35.9%)	2.229 (1.646 – 3.018)	<0.001*
Missing	32		Trend (< 0.001*)
Neonatal Complication:			
Intensive/Special care admission			
Normal weight	117 (11.3%)	Reference	
Underweight	15 (9.1%)	0.590 (0.296 – 1.176)	0.134
Overweight	67 (15.9%)	1.339 (0.921 – 1.947)	0.126
Obesity	73 (20.6%)	1.839 (1.269 – 2.665)	0.001*
Missing	36		Trend (<0.001*)
Fetal distress			
Normal weight	62 (6.7%)	Reference	
Underweight	17 (11.3%)	2.178 (1.147 – 4.133)	0.017*
Overweight	24 (6.0%)	1.222 (0.706 – 2.113)	0.474
Obesity	33 (9.6%)	1.870 (1.103 – 3.169)	0.020*
Missing	191		Trend (0.143)
Jaundice			
Normal weight	39 (4.8%)	Reference	
Underweight	11 (8.2%)	1.782 (0.821 – 3.866)	0.144
Overweight	30 (8.4%)	1.762 (1.017 – 3.053)	0.044*
Obesity	22 (6.8%)	1.592 (0.882 – 2.872)	0.123
Missing	382		Trend (0.163)
Respiratory distress			
Normal weight	38 (4.7%)	Reference	
Underweight	6 (4.5%)	0.879 (0.332 – 2.330)	0.796
Overweight	20 (5.6%)	1.275 (0.700 – 2.322)	0.427
Obesity	22 (6.8%)	1.294 (0.700 – 2.394)	0.411
Missing	382		Trend (0.288)
Congenital anomaly			
Normal weight	62 (7.3%)	Reference	
Underweight	10 (7.3%)	1.362 (0.662 – 2.804)	0.401
Overweight	26 (7.1%)	0.966 (0.559 – 1.668)	0.901
Obesity	25 (7.9%)	1.179 (0.692 – 2.011)	0.544
Missing	343		Trend (0.868)
Macrosomia (birthweight >4kg)			
Normal weight	140 (13.6%)	Reference	
Underweight	10 (6.2%)	0.389 (0.176 – 0.859)	0.019*
Overweight	70 (16.5%)	1.334 (0.945 – 1.884)	0.101
Obesity	93 (26.3%)	2.262 (1.619 – 3.160)	< 0.001*
Missing	43		Trend (< 0.001*)
Resuscitation			
Normal weight	253 (26.1%)	Reference	
Underweight	32 (20.9%)	0.675 (0.417 – 1.091)	0.108
Overweight	112 (27.8%)	1.186 (0.887 – 1.587)	0.248
Obesity	118 (34.5%)	1.499 (1.109 – 2.024)	0.009*
Missing	143		Trend (0.001*)

^*^Significant at the 0.05 level.

^a^Adjusted for maternal age, employment (categorical) and education, marital status (categorical), paternal employment (categorical) and education, household ownership, number of children under 16, alcohol intake and smoking status during pregnancy, maternal non-medical drug use, primiparity, pre-existing hypertension, and year of recruitment.

^b^Due to differences in hospital perinatal data collection among participating hospitals, some maternal morbidities have more missing data.

^c^ *p*-value for linear trend of adjusted ORs.

Mothers who completed and returned the 12-month follow-up questionnaire (63.9% of 2231) were more likely to be born in Australia (*p* = 0.013), married (*p* < 0.001), older (*p* < 0.001), employed (*p* < 0.001), had lower likelihood of psychological distress based on K6 scale (*p* < 0.001), consumed alcohol during pregnancy (*p* < 0.001), had higher education level (*p* < 0.001) and household income (*p* < 0.001), than those who did not return questionnaires. Further, they were less likely to have underweight or obese prepregnancy BMI (*p* = 0.009), or smoke during pregnancy (*p* < 0.001). There were no significant differences in gender of the babies.

### Outcomes at 12-month follow-up

The 12-month follow-up descriptive characteristics of participants and their babies are given in Table 4. There was a moderate negative ordinal relationship (-0.219, *p* < 0.001) between breastfeeding duration and prepregnancy BMI, indicating that the proportion of mothers who breastfed longer than or equal to 3 months decreased when prepregnancy BMI increased. Further, compared to women with a normal prepregnancy BMI, overweight, obese and underweight women were less likely to attend a play group or other recreational activity with their babies (*p* = 0.002) but are more likely to have medium or high risks of psychological distress (*p* = 0.032) and more frequently a lone parent (*p* = 0.030). A significant difference in weight retention at 12 months was observed between the four prepregnancy BMI groups (*p* < 0.001). There was a moderate positive ordinal relationship (0.221, *p* = 0.004) between asthma and prepregnancy BMI, indicating that the proportion of babies with asthma increases when prepregnancy BMI increases. The difference in weight gain of infants between normal and overweight mothers prepregnancy was also significant (*p* = 0.019).

**Table 4:** **12-month follow-up descriptive characteristics stratified by prepregnancy BMI (n = 1316)**

	Prepregnancy BMI				
	Underweight	Normal weight	Overweight	Obese	
	(BMI < 18.5)	(BMI 18.5-24.99)	(BMI 25-29.99)	(BMI ≥ 30)	
Characteristics	(N=103)	(N= 713)	(N= 289)	(N= 211)	***p***-value (two-sided)
Maternal Factor:					
Maternal mental health					
Low risk	87 (85.3%)	653 (92.2%)	256 (89.8%)	184 (87.6%)	0.032*^b^
Medium risk	11 (10.8%)	45 (6.4%)	27 (9.5%)	24 (11.4%)	
High risk	4 (3.9%)	10 (1.4%)	2 (0.7%)	2 (1.0%)	
Missing data	1	5	4	1	
5+ alcoholic drinks on one occasion (one per week or more often)	5 (4.9%)	54 (7.6%)	25 (8.7%)	8 (3.8%)	0.126
Missing data	0	0	2	0	
One month or more as a lone parent	22 (21.8%)	108 (15.3%)	39 (13.6%)	46 (21.8%)	0.030*^b^
Missing data	2	5	2	0	
Weight retention					
Mean in kg (SD)	3.64 (5.7)	2.33 (5.2)	2.48 (6.2)	-0.78 (9.3)	<0.001*^c^
Nutrition and Physical Activity Factor:					
Breastfeed longer than or equal to 3 months	69 (67.0%)	514 (72.7%)	171 (59.8%	116 (55.8%)	<0.001*^a-^
Missing data	0	6	3	3	
Four serves or more of fruit and vegetables each day	29 (28.2%)	230 (32.3%)	71 (24.6%)	56 (26.7%)	0.073
Missing data	0	1	0	1	
Takeaway food (more than once a week)	12 (11.8%)	49 (6.7%)	23 (8.0%)	26 (12.3%)	0.035*^b^
Missing data	1	9	1	0	
Play group or recreational activity with baby	53 (51.5%)	468 (65.7%)	158 (55.1%)	127 (60.5%)	0.002*^b^
Missing data	0	1	2	1	
Moderate physical activity (30+ minutes, at least once per week)	46 (44.7%)	443 (62.8%)	175 (61.2%)	128 (61.5%)	0.006*^b^
Missing data	0	8	3	3	
Walking (30+ minutes, at least once per week)	84 (81.6%)	634 (89.3%)	252 (88.4%)	175 (83.7%)	0.038*^b^
Missing data	0	3	4	2	
Child Development and Household Factor:					
Injury of child	19 (18.4%)	106 (14.9%)	50 (17.4%)	35 (16.7%)	0.658
Missing data	0	0	1	1	
Asthma	4 (3.9%)	59 (8.4%)	34 (11.8%)	26 (12.4%)	0.004*^a+^
Chest infection	17 (16.7%)	153 (21.7%)	63 (21.9%)	54 (25.7%)	0.330
Missing data	1	7	1	1	
Baby weight gain					
Mean in kg (SD)	6.38 (1.7)	6.34 (1.5)	6.64 (1.5)	6.62 (1.3)	0.019*^c^
Passive smoking	6 (5.8%)	40 (5.6%)	22 (7.7%)	15 (7.1%)	0.626
Missing data	0	0	2	0	

^*^Significant at the 0.05 level.

^a^Test for ordinal association using Gamma coefficient (^+^indicates a positive association; ^-^indicates a negative association).

^b^Test for association using chi-square test.

^c^Test for difference in means using ANOVA (Difference found between normal and overweight).

### Postpartum weight retention

In Table 5, the determinants of high postpartum weight retention (within the top quintile) for the normal, overweight, and obese prepregnancy BMI groups, with adjustments for potential confounding factors that are significantly associated with the prepregnancy BMI groups, are presented. Women were more likely to have high postpartum weight retention if they consumed three or less serves of fruit/vegetables (adjusted OR = 2.005, *p* = 0.001), did not engage in recreational activity with their babies (adjusted OR = 1.916, *p* < 0.001), spent less than once a week on walking for 30 minutes or more (adjusted OR = 1.691, *p* = 0.029) or spent time with friends less than once per week (adjusted OR = 1.695, *p* = 0.040). However, mothers who breastfed for more than 3 months had reduced the chance of high postpartum weight retention (adjusted OR = 0.673, *p* = 0.030).

**Table 5:** **Determinants of high postpartum weight retention (within the top quintile) with underweight prepregnancy BMI group excluded (n = 1213)**

Maternal and nutrition factor	Adjusted OR ^a^ (95% CI)	***p***-value (2-sided)
Breastfeed (≥3 months)	0.673 (0.471 – 0.961)	0.030*
Three or less serves of fruit/vegetables	2.005 (1.317 – 3.053)	0.001*
Takeaway foods (more than once a week)	1.006 (0.541 – 1.871)	0.986
No recreational activity with baby	1.916 (1.345 – 2.728)	< 0.001*
Moderate physical activity (less than once a week)	1.157 (0.815 – 1.641)	0.415
Walking - 30 mins or more (less than once a week)	1.691 (1.055 – 2.709)	0.029*
Maternal mental health (medium/high risk)	0.921 (0.523 – 1.624)	0.777
Live with biological father/spouse/partner	0.933 (0.498 – 1.748)	0.830
Have relationship status changed	0.788 (0.446 – 1.389)	0.410
One month or more as a single parent	0.876 (0.510 – 1.505)	0.633
Have a paid job after birth	0.966 (0.680 – 1.370)	0.845
Spend time with family (less than once a week)	0.874 (0.551 – 1.388)	0.569
Spend time with friends (less than once a week)	1.695 (1.024 – 2.804)	0.040*

^*^Significant at the 0.05 level.

^a^Adjusted for confounding factors: maternal age, employment (categorical) and education, marital status (categorical), paternal employment (categorical) and education, household ownership, number of children under 16, alcohol intake and smoking status during pregnancy, maternal non-medical drug use, primiparity, pre-existing hypertension, year of recruitment, and prepregnancy BMI grouping.

## Discussion

An important contributing factor to the development of long-term obesity in women is excessive weight retention after pregnancy [17, 19]. There is marked variability in weight changes that are associated with pregnancy and pregnancy is a critical period for prevention of obesity in women [35, 36]. This large cohort study has contributed to the growing evidence that helps to target interventions during pregnancy by the identification of modifiable risk factors that are associated with high postpartum weight retention. Women who consumed three or fewer serves of fruit/vegetables per day, did not engaged in recreational activity with their baby, and spending less time on leisure walking or with friends were at higher risk. Mothers who breastfed for more than 3 months had a lower likelihood of sustained weight retention after pregnancy. These findings help to clarify the inconsistent results obtained in previous studies [37, 38] on the role of dietary intake and breastfeeding duration as predictors of excessive weight retention postpartum, suggesting that the promotion of healthy eating, breastfeeding, modest physical activity and recreational activity with their baby and friends may help to improve weight retention outcomes. In this context, a systematic review of interventions designed to prevent excessive weight gain during pregnancy and postpartum weight retention found that there are only a few studies that evaluated the effect of interventions on postpartum weight retention and these interventions had limited success [39]. More recent randomized controlled trial interventions have been conducted aiming to reduce postpartum weight retention through nutritional counselling, exercise sessions, and psychological counselling [39, 40].

The prevalence of overweight and obesity prepregnancy in the present cohort of pregnant women in Southeast Queensland was 39%. Specifically, women with lower socioeconomic status, and adverse dietary habits appear to be at greater risk of being overweight or obese prepregnancy. For example, obese women were more likely to use non-medical drugs, smoke during pregnancy, have lower education level and their partners being unemployed or not in the labour force. They were less likely own their own home. More than half of them did not take major supplements prior to and during pregnancy. These findings have contributed to the evidence-base obtained by studies conducted in various countries [24, 25, 41] on the association of socioeconomic status with pregnancy obesity or high postpartum weight retention. In addition, overweight and obese women are less likely to breastfeed longer than 3 months, partly due to that obese women are less likely to express milk successfully within 2 months postpartum [42], and engage in recreational activities with their baby. This high-risk subgroup of women may therefore greatly benefit from interventions to improve weight retention outcomes by promoting nutrition knowledge, healthy eating and physical activity. The present study also confirms that overweight and obese women are more likely to have medium or high risks of psychological distress and more frequently identify as a lone parent. This finding suggests that interventions that incorporate counselling and support with psychologically-based components, such as monitoring of maternal depression and anxiety, may provide an effective way to prevent excessive gestational weight gain [39].

Women who enter pregnancy overweight or obese have been associated with many adverse maternal and neonatal outcomes, based on studies in various countries [9–11]. The current study showed that prepregnancy obese women, who comprised 18% of the cohort, had a significantly increased risk of obstetric complications including gestational diabetes, preeclampsia, cesarean delivery, and their newborns had increased risk of poor neonatal outcomes including intensive or special care nursery admission, fetal distress, resuscitation, and macrosomia. In prepregnancy overweight women, who comprised 21% of the cohort, increased risk was associated with cesarean delivery and jaundice. This evidence has important public health implications. It would assist the health care provider to identify at-risk pregnancies, potential obstetric complications, and to monitor the newborns during the immediate post-delivery period.

### Strengths and limitations

The key strength of this study was the large cohort, the population-based recruitment, and the prospective nature of the data collection. This large cohort enables the inclusion of a wide variety of health-related exposures and outcomes in the analysis and hence an increased validity and generalisability of our findings can be achieved. Besides maternal, pregnancy and neonatal characteristics, the study design allowed risk factors in the domains of nutrition/physical activity and household/family structure to be considered. However, careful consideration should be given to whether these factors may be on the causal pathway between the exposure and the outcome. Adjusting for these "pathway" variables in the models may induce bias in the estimated effect of an exposure [43]. While there are differences in some household/family factors between prepregnancy BMI groups, only household ownership and number of children under 16 are confounders and were adjusted for in the multivariate analyses. Notwithstanding these overall strengths the results should be interpreted within the context of the following study limitations. Despite the population-based recruitment, the EFHL cohort, as demonstrated in Table 1, is likely to include fewer mothers under 20 years of age, and fewer low birth-weight and low gestational age babies than in the general population. This is because prospective mothers were recruited after 24 weeks gestation at routine clinic visits, with a significant proportion of women recruited closer to 36 weeks [27]. This implies that mothers who are at risk for childbirth complications or adverse birth outcomes could be underrepresented in our sample. Due to differences in hospital perinatal data collection among the participating hospitals, only common maternal morbidities were included in the data analyses. Among them, gestational diabetes and preeclampsia are rare (less than 5%) in our cohort, which mean that adjusted ORs for these two outcomes cannot be obtained for the underweight group (see Table 3). While a wide variety of confounding factors have been considered in the study, there may still be residual confounding present, resulting in distortions in the estimated effect size of exposures.

As with most cohort studies, a degree of measurement error could be expected arising from self- or proxy-administered nature of the survey data collection that could affected the accuracy of the self-reported prepregnancy and postpartum weights variables used in this study. Given however, adult weight and BMI are underestimated by self-reported measures, especially in overweight and obese groups [44], it is likely the true prevalence of overweight and obese pregnant women may be higher than found in our cohort. Loss to follow up bias may also have resulted in an underestimation of postpartum weight retention and the prevalence of adverse 1-year maternal and child outcomes. With respect to the potential selection bias and loss of follow-up bias in this study, there was an overrepresentation of relatively advantaged mothers who agreed to participate in the cohort study and who provided 1-year follow-up information. These women had higher education levels and household income but were less likely to have prepregnancy obesity.

### Implications for practice and/or policy

Compared to the extensive body of literature on the consequences of obesity in non-pregnant individuals [45–48], research to date on the impact of obesity on reproductive outcomes is relatively scant, particularly population-based studies on extremes of BMI [5]. However, the importance of this problem is beginning to receive considerable attention [49]. The contribution of this paper to the growing literature is in its robust findings across primary, secondary and tertiary components of the problem. The paper documents in a population-based cohort of pregnancy women, the distribution and determinants of overweight and obesity, the likely perinatal complications of the condition and the distribution and determinants of maternal and child outcomes 12 months after delivery including postpartum weight retention.

## Conclusions

The reported findings provide additional specificity to the increasing evidence of the predisposition of obesity prepregnancy on adverse maternal and perinatal outcomes. They also offer a coherent picture that both justifies the need for large scale interventions to address the problem, but also the information about risk factors and risk groups that is required to support the development and implementation of these interventions. Future research and practice could usefully now be addressed at evaluating the efficacy and population level effectiveness of the anticipated policy solutions, to what is clearly a maternal and child health problem of major proportions.

## Authors’ information

Shu-Kay Ng, PhD, is Associate Professor of Biostatistics at Griffith University. He is the primary statistician for the EFHL study. His research focuses on longitudinal research and the epidemiology of obesity, birth outcomes and socioeconomic disparities.

Cate Cameron, PhD, is a Senior Research Fellow funded by the NHMRC (ID428254) and founding investigator of EFHL. She is an epidemiologist with a focus on longitudinal research, data linkage, maternal and child health outcomes.

Andrew Hills, PhD, is Professor of Allied Health Research – Maternity and Neonatology and Director – Centre for Nutrition and Exercise at Mater Research. His research focuses on obesity in mothers and offspring, particularly body composition assessment.

Rod McClure has medical qualifications, clinical experience in emergency medicine, a PhD in injury epidemiology and specialist training in public health medicine. Rod McClure is currently the Director of the Injury Research Institute, Monash University.

Paul Scuffham, PhD, is Professor of Health Economics, Director of Population & Social Health Research at Griffith University, and leads the EFHL study. His research focuses on assessing value for money in healthcare and consumer engagement in health policy decisions.

## Abbreviations

BMI: Body mass index
EFHL: Environments for Healthy Living
GDM: Gestational diabetes mellitus
K6: Kessler 6
WHO: World Health Organisation
ANOVA: Analysis of variance
OR: Odds ratio
CI: Confidence interval.
